# Association between BNT162b2 vaccination and health-related quality of life up to 18 months post-SARS-CoV-2 infection in Israel

**DOI:** 10.1038/s41598-023-43058-1

**Published:** 2023-09-22

**Authors:** Paul Kuodi, Yanay Gorelik, Hiba Zayyad, Ofir Wertheim, Karine Beiruti Wiegler, Kamal Abu Jabal, Amiel A. Dror, Jelte Elsinga, Saleh Nazzal, Daniel Glikman, Michael Edelstein

**Affiliations:** 1 Azrieli Faculty of Medicine, Bar-Ilan University, Safed, Israel; 2 Ziv Medical Centre, Safed, Israel; 3 Baruch Padeh Medical Centre, Poriya, Israel; 4 Galilee Medical Centre, Nahariyah, Israel; 5 Amsterdam University Medical Centre, Amsterdam, The Netherlands

**Keywords:** Infectious diseases, Quality of life, Epidemiology

## Abstract

We determined whether COVID-19 vaccination was associated with Quality of Life (QoL) changes among individuals previously infected with SARS-CoV-2 in Israel. Using a validated questionnaire, we collected information about socio-demographics, SARS-CoV-2 infection, COVID-19 vaccination and QoL (using the EQ-5D-5L tool) 3–18 months post-infection among adults tested for SARS-CoV-2 by polymerase chain reaction in Northern Israel between March 2020–June 2022. We compared post-COVID QoL between those vaccinated against COVID-19 at the time of infection and those not, using an adjusted linear regression model, stratified by time elapsed since infection. Of 951 participants, mean EQ-5D Utility Index (EQ-5D UI) was 0.82 (SD = 0.26) and 0.83 (SD = 0.25) among the 227 double and 250 triple vaccinated respectively, compared to 0.76 (SD = 0.33) among those who received 0 dose (*n* = 243). The size of the effect of vaccination was small (Cohen’s d = 0.2). In the adjusted model, previously infected individuals vaccinated with two or more doses reported a QoL score post- infection 0.05 points higher (CI = 0.01–0.10, *p* = 0.02) compared with those unvaccinated when infected. No association between vaccination and QoL was detected beyond 12 months post-infection. Vaccination with two or more doses of COVID19 vaccine, or at least the BNT162b2 vaccine, may modestly mitigate QoL losses associated with post-acute COVID-19 symptoms, at least in the first 12 months post-infection.

## Introduction

Post-COVID condition, also referred to as post-acute sequelae of COVID-19 (PASC) or Long COVID was defined by the World Health Organization (WHO) as “a condition that occurs in individuals with a history of probable or confirmed SARS-CoV-2 infection and occurs three months after the initial COVID-19 symptoms, whereby the symptoms reported by post-COVID patients cannot be explained by alternative diagnoses”^1^. PASC affects approximately 10–30% of individuals previously infected with SARS-CoV-2^2^. Suggested pathophysiological mechanisms for PASC include long-term organ damage resulting from the initial infection, central nervous system damage, immune dysregulation, endothelial dysfunction, viral persistence, and coagulation activation^3, 4^. It has also been postulated that several pathological mechanisms may occur concurrently, manifesting as a wide range of seemingly unrelated post-COVID symptoms^3^ and that the patient experience of long COVID results from the interplay between biological, social, psychological and experiential factors^5^.

Post-SARS-CoV-2 infection persistence of symptoms is a widely reported phenomenon in the literature. A living systematic review (ongoing as of January 2023) suggested that severity of the acute episode was associated with post-COVID condition^6^. Chronic conditions such as diabetes, hypertension, Parkinson’s disease, chronic obstructive pulmonary disease and others were also associated with developing post-COVID condition^7^. Post-Covid condition has also been reported among asymptomatic COVID19 patients, albeit to a lesser extent than symptomatic individuals^7^. Post-viral syndrome is not unique to SARS-CoV-2 and has been described with other viral infections including Middle East Respiratory Syndrome (MERS) and Severe Acute Respiratory Syndrome (SARS)^8^.

Mass COVID-19 vaccination has been one of the key measures to mitigate the impact of the pandemic. Despite decreased effectiveness against infection with Omicron sub-variants, COVID-19 vaccines remain effective against severe disease^9^. By December 2022, approximately 70% of the world population and 71% of the Israeli population had received at least one dose of a COVID-19 vaccine^10, 11^. Israel overwhelmingly used the BNT162b2 mRNA vaccine. As of February 2023, Israel offers up to 5 doses of COVID-19 vaccine, including an updated bivalent vaccine used as a booster that has shown efficacy against severe disease caused by the Omicron variants of SARS-CoV-2^12^.

Beyond protecting against acute COVID-19, being vaccinated at the time of infection is also associated with a reduction in reported post-acute symptoms, with most studies published on the topic agreeing on the direction of the association, if not the strength of the effect^13–15^. The evidence of post-infection vaccination on long-term symptoms is less clear^16^. Available evidence suggests that post-acute COVID-19 symptoms impacts quality of life (QoL)^17, 18^, although the extent of the impact on QoL depends on factors such as acute disease severity^19^ and gender^17, 20^ The emerging consensus is that QoL impairments sustained during acute-COVID-19 persists for months as a result of ongoing physical and mental health issues^17, 20^. Despite the growing consensus around the mitigating effect of COVID19 vaccinations against post-acute symptoms and the impact of post-acute COVID symptoms on QoL, there is a gap in evidence on whether COVID-19 vaccination has the potential to mitigate any QoL losses among SARS-CoV-2 infected individuals suffering from long-term symptoms.

Understanding the impact of vaccination on long-term QoL resulting from post-acute COVID-19 symptoms will help estimate the global burden of disease likely to emerge as a result of the COVID-19 pandemic, and better define the role of vaccination in mitigating it. We therefore aimed to identify associations between COVID-19 vaccination and QoL among individuals previously infected with SARS-CoV-2, up to 18 months after infection by comparing QoL between vaccinated and unvaccinated individuals.

## Results

### Baseline characteristics

Between July 2021 and June 2022, 95,604 persons were invited to participate in the study. Of these, 6964 (7.3%) individuals responded and provided complete data on their COVID-19 vaccination and SARS-CoV-2 RT-PCR testing status and were thus included in the study. Of the 6,964 included participants, 2579 (37.0%) participants reported a positive test. Of those, 1,227 (47.6%) participants reported complete information about post-COVID symptoms and QoL. Of these, 276 (22.5%) individuals reported their symptoms less than 60 days following their positive PCR test and were excluded from the study so as to not include the impact of acute illness on QoL. The remaining 951 participants were included in the final analysis. The baseline socio-demographic characteristics of the study participants are shown in Table 1. Overall, mean (SD) age was 46 (± 14.74) years old, 65.7% of participants were female, and 76.9% were of Jewish ethnicity, comparable to the 74% in the general population^21^. Compared to infected participants not included in the study (because they did not have complete QoL data), participants were similar in terms of age (mean 46 vs 49 years old, *p* = 0.88), gender (34 vs 39% male, *p* = 0.06), vaccination status (50.1 vs 53.1% who received at least 2 doses, *p* = 0.26) and severity of disease (14.3% vs 12.2% hospitalized, *p* = 0.23). In terms of vaccination status, of the 951 participants, 243 participants (25.6%) were unvaccinated, and 231 (24.3%), 227 (23.9%) and 250 (26.2%) received 1, 2 and 3 doses of COVID-19 vaccine respectively. Vaccinated participants were comparable with unvaccinated participants with respect to gender and marital status. The unvaccinated were more likely to be hospitalized for COVID-19 and slightly younger than those vaccinated (44.3 vs. 47.9 years, *p* < 0.001), likely reflecting the fact that vaccination in Israel was first available to older individuals. In the unvaccinated group, the mean duration between reporting testing positive for SARS-CoV-2 and answering the survey was 251 days compared to 401, 267 and 137 days for 1-dose, 2-doses, and 3-doses vaccinated, respectively (Table 1). The longer follow-up time for those who received one dose reflects the fact that the vast majority (206/231, 89.2%) of those who received a single dose were infected prior to vaccination, as the policy in Israel was initially for those infected to received single dose of vaccine. Of the 951 participants, 572 (60.1%) reported at least one post-COVID symptom and 547 (57.5%) one of the ten most common symptoms (listed in supplementary Table s1). Of the 547 participants reporting symptoms, 298 had received 0 or 1 vaccine dose and 127 and 147 had received 2 and 3 doses respectively.

**Table 1:** Socio-demographic and clinical characteristics of participants.

Variables	Number of participants with available information	All participants	Unvaccinated	One Dose	Two Doses	Three Doses	*p*-value
		951	243	231	227	250	
Age (Mean (SD))	951	46.0 (14.7)	44.6 (15.2)	43.4 (12.8)	48.3 (15.4)	47.7 (15.0)	< 0.001
Age group (*n*, %)	951						0.002
> 60		178 (18.7)	45 (18.5)	25 (10.8)	53 (23.3)	55 (22.0)	
18–40		387 (40.7)	111 (45.7)	106 (45.9)	74 (32.6)	96 (38.4)	
41–60		386 (40.6)	87 (35.8)	100 (43.3)	100 (44.1)	99 (39.6)	
Sex (*n*, %)	938						
Male (%)		322 (34.3)	88 (36.8)	80 (35.1)	76 (34.4)	78 (31.2)	0.614
Marital status (*n*, %)	763						
Single (%)		176 (23.1)	48 (23.8)	49 (26.2)	40 (22.5)	39 (19.9)	0.525
Education (n, %)	611						0.070
Elementary school		71 (11.6)	18 (12.7)	24 (15.3)	15 (10.1)	14 (8.6)	
High school		64 (10.5)	25 (17.6)	12 (7.6)	15 (10.1)	12 (7.4)	
Postgraduate		162 (26.5)	33 (23.2)	40 (25.5)	39 (26.2)	50 (30.7)	
Undergraduate		314 (51.4)	66 (46.5)	81 (51.6)	80 (53.7)	87 (53.4)	
Ethnicity (*n*, %)	762						
Non-Jewish		247 (32.4)	70 (34.7)	90 (48.1)	41 (23.0)	46 (23.6)	< 0.001
Hospitalized (*n*, %)	893						
Yes		128 (14.3)	47 (20.9)	34 (16.8)	30 (13.9)	17 (6.8)	< 0.001
ICU admission (*n*, %)	951						
Yes		33 (28.0)	17 (38.6)	7 (22.6)	6 (22.2)	3 (18.8)	< 0.001
Diabetes (*n*, %)	951						
Yes		47 (4.9)	5 (2.1)	12 (5.2)	15 (6.6)	15 (6.0)	0.099
Hypertension (*n*, %)	951						
Yes		100 (10.5)	15 (6.2)	19 (8.2)	32 (14.1)	34 (13.6)	0.008
Asthma (*n*, %)	951						
Yes		34 (3.6)	7 (2.9)	7 (3.0)	10 (4.4)	10 (4.0)	0.771
COPD (*n*, %)	951						
Yes		9 (0.9)	2 (0.8)	3 (1.3)	2 (0.9)	2 (0.8)	0.938
Chronic kidney disease (*n*, %)	951						
Yes		6 (0.6)	2 (0.8)	0 (0.0)	2 (0.9)	2 (0.8)	0.583
Days followed (Mean (SD))	951	261 (188)	251 (171)	401 (148)	266 (189)	137 (141	< 0.001
Time since infection (*n*, %)	951						< 0.001
3–6 months		443 (46.6)	105 (43.2)	13 (5.6)	108 (47.6)	217 (86.8)	
7–12 months		234 (24.6)	83 (34.2)	89 (38.5)	48 (21.1)	14 (5.6)	
> 12 months		274 (28.8)	55 (22.6)	129 (55.8)	71 (31.3)	19 (7.6)	

*COPD*: Chronic obstructive pulmonary disease, *N*: Number of participants responding per variable.

### QoL and post-COVID symptoms

#### EQ-5D-5L dimensions

**Figure 1:**
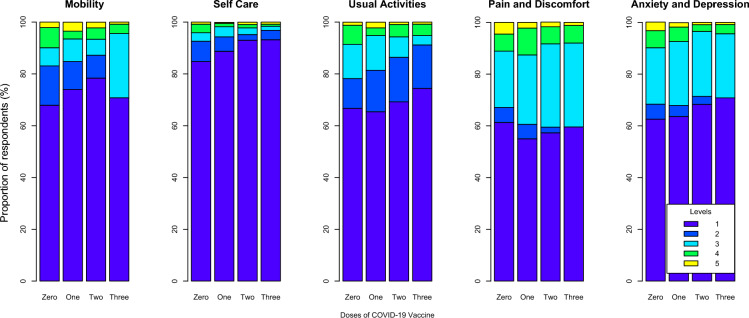
Proportions of participants reporting each level of EQ-5D-5L dimensions.

Compared with 2 and 3-dose vaccinated participants, a higher proportion of unvaccinated and one-dose vaccinated participants reported scores of 4 and 5 (indicating a lower QoL) in the mobility, pain, discomfort, and anxiety and depression dimensions of EQ-5D-5L, a standardized Quality of life questionnaire collecting information on 5 dimensions of quality of life, each with a score between 1 (lowest quality of life) and 5 (highest) (Fig. 1, supplementary Table s2). The proportion of individuals reporting no or only slight impairment in their usual activities was higher among those who received two or three doses compared with those who received 0 or 1 dose for the self-care, usual activities, and anxiety and depression dimensions (Fig. 1 and supplementary Table s2).

#### EQ-5D utility index (UI) scores

**Figure 2:**
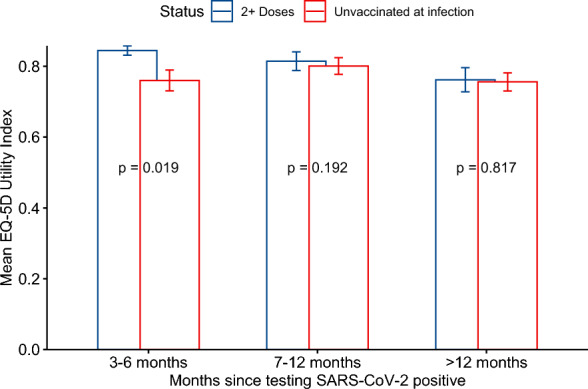
Crude utility indexes for vaccinated participants and for participants not vaccinated at the time of infection according to duration since SARS-CoV-2 testing (error bars denotes 95% confidence intervals).

Regardless of their COVID-19 vaccination status, participants not reporting post-acute COVID-19 symptoms had mean EQ-5D UI of 0.92 (SD = 0.20), compared to 0.72 (SD = 0.32) among individuals reporting at least one symptom 3–18 months post COVID. There was no effect of vaccination on QoL among those not reporting post-acute symptoms (Cohen’s d = − 0.06). Overall, the mean EQ-5D UI was 0.82 (SD = 0.26) and 0.83 (SD = 0.25) among the double and triple vaccinated respectively, compared to 0.76 (SD = 0.33) and 0.78 (SD = 0.31) among those who had received 0 or 1 dose, respectively (Table 2). The overall size of the effect of being vaccinated at the time of infection was small (Cohen’s d = 0.2). Among participants reporting at least one post-COVID symptom, those unvaccinated had a mean EQ-5D UI of 0.68 (SD = 0.40) compared with 0.74 (SD = 0.27) and 0.77 (SD = 0.27) for those doubly and triply vaccinated, respectively, a small effect size (Cohen’s d of 0.22 and 0.32 respectively). In all age, gender, and ethnicity subgroups, the double and triple-vaccinated individuals reported higher mean UIs compared to the unvaccinated, with small effect sizes in all strata (Cohen’s *d* < 0.5, Table 2). The largest effect size of vaccination on QoL was seen among individuals aged 60 and over (Cohen’s d = 0.4). By time since infection those vaccinated reported higher unadjusted UIs compared to those unvaccinated (0-doses and 1-dose) at 3–6 months, (0.84 ± 0.24 vs. 0.76 ± 0.32, *p* = 0.019, Cohen’s d = 0.3, Fig. 2). No overall significant difference in UI was found according to vaccination status among those reporting 7–12 months or more than 12 months after their acute SARS-CoV-2 infection.

**Table 2:** Crude mean utility indexes among participants according to baseline characteristics and vaccination status.

Variables		Overall (vaccinated + unvaccinated at infection)			Stratified by number of vaccine doses received				Standardized mean difference (Cohen’s d) between those vaccinated at the time of infection (2 + doses) and those not (0 and 1 dose)
		*n*	Mean	SD	Covid vaccine doses	n	Mean	SD	
Overall		951	0.8	0.29	0-Doses	243	0.76	0.33	0.2
					1-Dose	231	0.78	0.31	
					2-Doses	227	0.82	0.26	
					3-Doses	250	0.83	0.25	
Age	18–40	387	0.84	0.25	0-Doses	111	0.81	0.29	0.03
					1-Dose	106	0.85	0.18	
					2-Doses	74	0.86	0.22	
					3-Doses	96	0.82	0.26	
	41–60	386	0.77	0.31	0-Doses	87	0.744	0.34	0.24
					1-Dose	100	0.73	0.37	
					2-Doses	100	0.77	0.3	
					3-Doses	99	0.83	0.24	
	> 60	178	0.78	0.33	0-Doses	45	0.67	0.43	0.41
					1-Dose	25	0.69	0.42	
					2-Doses	53	0.85	0.22	
					3-Doses	55	0.84	0.24	
Sex	Female	616	0.79	0.28	0-Doses	151	0.76	0.32	0.18
					1-Dose	148	0.77	0.29	
					2-Doses	145	0.80	0.28	
					3-Doses	172	0.83	0.23	
	Male	322	0.81	0.31	0-Doses	88	0.76	0.38	0.26
					1-Dose	80	0.79	0.34	
					2-Doses	78	0.86	0.22	
					3-Doses	76	0.83	0.27	
Ethnicity	Jewish	515	0.81	0.28	0-Doses	132	0.76	0.36	0.12
					1-Dose	97	0.84	0.25	
					2-Doses	137	0.81	0.26	
					3-Doses	149	0.84	0.22	
	Others	247	0.75	0.33	0-Doses	70	0.75	0.33	0.26
					1-Dose	90	0.69	0.37	
					2-Doses	41	0.84	0.23	
					3-Doses	46	0.74	0.32	
Post Covid symptoms	Asymptomatic	379	0.92	0.20	0-Doses	92	0.93	0.17	− 0.06
					1-Dose	84	0.92	0.21	
					2-Doses	100	0.91	0.21	
					3-Doses	103	0.92	0.18	
	Symptomatic	572	0.72	0.32	0-Doses	151	0.66	0.38	0.28
					1-Dose	147	0.70	0.33	
					2-Doses	127	0.74	0.27	
					3-Doses	147	0.77	0.27	
Months since SARS-CoV-2 testing	3 to 6 months	443	0.82	0.26	0-Doses	105	0.78	0.32	0.35
					1-Dose	13	0.63	0.27	
					2-Doses	108	0.84	0.24	
					3-Doses	217	0.85	0.24	
	7 to 12 months	234	0.80	0.28	0-Doses	83	0.76	0.35	0.07
					1-Dose	89	0.84	0.26	
					2-Doses	48	0.83	0.20	
					3-Doses	14	0.76	0.24	
	> 12 months	274	0.76	0.34	0-Doses	55	0.74	0.37	0.02
					1-Dose	129	0.76	0.34	
					2-Dose	71	0.77	0.32	
					3-Doses	19	0.73	0.35

### Association between COVID-19 vaccine and EQ‑5D‑5L UI and patient characteristics

After adjusting for age, ethnicity, hypertension, hospitalization (as a proxy for severity), and duration since testing positive for SARS-CoV-2), SARS-CoV-2-infected individuals vaccinated with 2 or 3 doses reported 0.05 points higher UI compared to those unvaccinated at the time of infection (95%CI = 0.01–0.10, *p* = 0.024, Table 3). Compared with those not vaccinated at the time of infection, the double-vaccinated reported an overall 0.06 points higher mean QoL score post-infection (95%CI = 0.004–0.11, *p* = 0.036, Table 3), but the mean UI in those triply-vaccinated was not significantly different (+ 0.05, 95%CI = -0.01–0.10, *p* = 0.096, Table 3). When hospitalization was removed from the model, we found that vaccination was associated with a 0.06 point (*p* = 0.011) higher UI among those vaccinated with two doses or more compared to those not (supplementary Table s3).

**Table 3:** Crude and adjusted changes in utility indices for SARS-CoV-2 infected participants (all participants and for participants experiencing post-COVID symptoms, 3–18 months).

Variables	Variables	All participants							Participants experiencing post-covid symptoms						
		n	Crude analysis			Adjusted			n	Crude analysis			Adjusted analysis		
			Change in utility score (percentage points)	95% CI	*p*	Change in utility score (percentage points)	95% CI	*p*		Change in utility score (percentage points)	95% CI	*p*	Change in utility score (percentage points)	95% CI	*p*
Sex	Male	322	Baseline						410						
	Female	616	0.02	− 0.06–0.02	0.396	− 0.08	− 0.13–− 0.04	** < 0**.**001**	154	0.04	− 0.02–0.10	0.147	− 0.06	− 0.13–0.01	0.071
Age (continuous)			< − 0.01	< − 0.01– < − 0.01	** < 0.001**	< − 0.01	< − 0.01– < 0.01	**0**.**059**	572	< 0.01	− 0.01 – < 0.01	** < 0**.**001**	< − 0.01	< − 0.01– < 0.01	**0**.**106**
Ethnicity	Jewish	515	Baseline						318						
	Other	247	− 0.07	− 0.11–− 0.02	**0**.**004**	− 0.05	− 0.10 – < 0.01	**0**.**032**	165	− 0.09	− 0.15–− 0.03	**0**.**004**	− 0.06	− 0.12–− 0.01	**0**.**048**
Vaccine doses	Unvaccinated at infection	474	Baseline						298						
	2 Doses	227	0.05	< − 0.01– 0.09	0.060	0.06	< 0.01–0.11	**0**.**036**	147	0.06	− 0.01–0.13	0.069	0.07	< − 0.01– 0.15	0.065
	3 Doses	250	0.06	0.01–0.10	**0**.**011**	0.05	− 0.01–0.10	0.096	127	0.09	0.03–0.15	**0**.**006**	0.09	0.02–0.16	**0**.**024**
	2 + 3 Doses	477	0.05	0.01–0.09	**0**.**007**	0.05	0.01–0.10	**0**.**024**	274	0.08	0.02–0.13	**0**.**005**	0.08	0.02–0.14	**0**.**013**
Days since SARS-CoV-2 positive (continuous)		951	< 0.01	< − 0.01– < 0.01	0.830	< 0.01	< − 0.01– < 0.01	0.828	572	< 0.01	< − 0.01– < 0.01	0.164	< 0.01	< − 0.01– < 0.01	0.914
Hypertension	No	851	Baseline						511						
	Yes	100	− 0.16	− 0.22–− 0.10	** < 0**.**001**	− 0.13	− 0.21–− 0.06	** < 0**.**001**	61	− 0.17	− 0.26–− 0.09	** < 0**.**001**	− 0.10	− 0.20–− 0.01	**0**.**032**
Hospitalisation (COVID-19 Severity)	No	765	Baseline						435						
	Yes	128	− 0.25	− 0.30–− 0.19	** < 0**.**001**	− 0.23	− 0.29–− 0.17	** < 0**.**001**	95	− 0.29	− 0.35–− 0.22	** < 0**.**001**	− 0.26	− 0.34–− 0.18	** < 0**.**001**

Significant values are in [bold].

When restricting the analysis to those experiencing ongoing post-COVID symptoms and after adjustment for potential confounders, participants who received two or three doses reported 0.08 higher UI compared to those unvaccinated at the time of infection (CI = 0.02–0.14, *p* = 0.013). When stratifying by the number of doses received, the association was only statistically significant with three doses (+ 0.09, 95%CI = 0.02–0.16, *p* = 0.024, Table 3). When removing hospitalization out of the model, elevation in UIs overall among the vaccinated compared to the unvaccinated at the time of infection were 0.08 (*p* = 0.012), 0.07 (*p* = 0.059) and 0.08 (*p* = 0.016) overall and for two and three or more doses respectively (supplementary Table s3).

### Association between COVID-19 vaccine and EQ‑5D‑5L UIs at different time points post-SARS-CoV-2 infection

Among participants who answered the survey between 3- and 6-months post-SARS-CoV-2 infection, before adjusting for confounders, those who received 2 or 3 doses of vaccine reported a 0.08-point higher QoL (95%CI 0.03–0.14, *p* < 0.003). The effect size was lower, and the association was no longer statistically significant after adjusting for confounders significant in the univariate analysis (+ 0.03, 95% CI = − 0.03–0.10, *p* = 0.303, Table 4). When adjusting for all confounders except hospitalization, vaccination was associated with a 0.07-point higher EQ-5D-UI (*p* = 0.036, supplementary Table s3). Conversely, when adjusting for hospitalization only, the association between vaccination and post-COVID QoL 3–6 months post-infection was not significant (+ 0.08 points, *p* = 0.303). Among those reporting 7–12 months post-infection, there was no overall association between COVID-19 vaccination and mean EQ‑5D‑5L UI, however among those reporting post-acute symptoms, double and triple vaccinated participants reported 0.15 higher mean UI compared to those unvaccinated at the time of infection (95% CI 0.02–0.29, *p* = 0.024, Table 4). We did not detect any association between COVID-19 vaccination and mean EQ‑5D‑5L UI among participants reporting beyond 12 months post-SARS-CoV-2 infection, whether taking acute disease severity into account or not (Table 4).

**Table 4:** Crude and adjusted changes in utility indices for SARS-CoV-2 infected participants for all participants and for participants experiencing post-COVID symptoms, by time elapsed since testing.

Time elapsed since testing	Variables	All participants							Participants experiencing post-covid symptoms						
	Vaccine doses	n	Univariate model			Adjusted			n	Crude analysis			Adjusted analysis		
			Change in utility score	95% CI	*p*	Change in utility score	95% CI	*p*		Change in utility score	95% CI	*p*	Change in utility score	95% CI	*p*
3–6 months	Unvaccinated at the time of infection	118	Baseline						80	Baseline					
	2-Doses	108	0.08	0.02–0.18	**0.017**	0.04	− 0.04–0.12	0.302	53	0.06	− 0.05–0.16	0.274	− 0.01	− 0.13–0.10	0.831
	3-Doses	217	0.09	0.03–0.14	**0**.**005**	0.03	− 0.04–0.10	0.396	128	0.11	0.02–0.19	**0**.**012**	0.05	− 0.05–0.14	0.325
	2 + 3 Doses	325	0.08	0.03–0.14	**0**.**003**	0.03	− 0.03–0.10	0.303	181	0.09	0.01–0.17	**0**.**021**	0.03	− 0.06–0.12	0.506
7–12 months	Unvaccinated at the time of infection	172	Baseline						109	Baseline					
	2-Doses	48	0.03	− 0.06–0.12	0.540	0.11	− 0.01–0.22	0.063	35	0.10	− 0.03–0.23	0.123	0.17	0.03–0.31	**0**.**017**
	3-Doses	14	− 0.04	− 0.19–0.12	0.639	0.06	− 0.12–0.24	0.529	9	− 0.01	− 0.22– 0.21	0.963	0.06	− 0.17–0.30	0.593
	2 + 3 Doses	62	0.01	− 0.07–0.10	0.746	0.10	− 0.01–0.20	**0**.**072**	44	0.07	− 0.04–0.19	0.187	0.15	0.02–0.29	**0**.**024**
> 12 months	Unvaccinated at the time of infection	184	Baseline						109	Baseline					
	2-Doses	71	0.01	− 0.08–0.11	0.765	0.06	− 0.06–0.17	0.348	39	0.04	− 0.09–0.17	0.505	0.05	− 0.07–0.16	0.410
	3-Doses	19	− 0.03	− 0.19–0.14	0.759	0.03	− 0.16–0.22	0.755	10	0.02	− 0.22–0.25	0.897	0.04	− 0.15–0.23	0.658
	2 + 3 Doses	90	0.01	− 0.08–0.09	0.892	0.05	− 0.06–0.16	0.362	49	0.04	− 0.08–0.16	0.530	0.05	− 0.06–0.16	0.390

Adjusted for: ethnicity, sex, age, hypertension, and hospitalization.

Significant values are in [bold].

## Discussion

To our knowledge, this is the first study that investigates the long-term impact of COVID-19 vaccination on QoL outcomes among individuals previously infected with SARS-CoV-2. After adjusting for potential confounders, we found that being vaccinated with 2 or more doses of COVID-19 vaccine at the time of infection was associated with higher QoL post-SARS-CoV-2 infection, more so among individuals experiencing post-COVID symptoms. The size of the effect identified was small and time limited. These results suggest that, overall, COVID-19 vaccination, or at least with the BNT162b2 vaccine widely used in Israel, may partly mitigate losses of QoL post-acute COVID-19, as measured by the EQ-5D-5L, at least in the first 12 months. The small effect size suggests that while vaccines should be considered as part of an array of tools and approaches to mitigate long COVID, it is not a silver bullet against the QoL life loss associated with post-acute COVID symptoms. We could not find a minimally clinically important difference (MCID) in EQ-5D for patients with post-viral symptoms. While evidence from other diseases suggest that a change in UI as small as 0.03 can be clinically important^22^,MCIDs are subjective and not easily transferrable from one clinical condition to another. MCIDs in EQ5D for post viral diseases are needed to interpret the clinical relevance of changes in QoL following interventions (vaccines or otherwise) intending to mitigate Long COVID. . In our cohort, the severity of the initial COVID-19 illness (measured by hospitalization) was the most important confounding factor associated with QoL in the post-COVID period. In the 3–6 months following acute infection, hospitalization explained most of the association between vaccination and QoL. This suggests that the well documented effectiveness of COVID-19 vaccination against severe acute disease,^9^ also impact of post-acute symptoms, since severity of acute disease is a strong predictor of post-acute COVID symptoms^23^. In other words, COVID-19 vaccination mitigates the loss of QoL associated with post-acute COVID symptoms by reducing the severity of the acute illness, which in turn reduces the likelihood and severity of ongoing, post-acute disease. Our results also suggest that this may not be the only mechanism of action: overall and among those reporting 7–12 months post-acute infection and reporting post-acute COVID symptoms, those triply vaccinated reported a higher QoL compared to those unvaccinated, even after adjusting for hospitalization. These findings suggest that even in instances where vaccinated patients report post-acute symptoms, and after taking disease severity into account, the impact of these symptoms on QoL is less than among those who are unvaccinated. There was no significant association between vaccination and QoL among those reporting 12 months or more post-infection. While we refrain from statistically analysing trends in UI over time because patients are different at each time point, this regression towards the UI of those unvaccinated suggests that the positive effect that vaccination may have on QoL may wane over time. This hypothesis should be tested more formally with longitudinal studies. Waning of COVID-19 vaccine effectiveness against reinfection and severity of symptoms of acute COVID-19 illness has been reported previously^24, 25^. Our findings suggest that booster doses may be required to offer continued mitigation against the post-acute effects of SARS-CoV-2 infection, although the data presented here cannot answer with any level of certainty whether this is the case.

To a large extent, the demographic characteristics of our study participants approximated that of the Israeli population in terms ethnicity and age distribution and reflected the national vaccine roll out strategy which targeted individuals older than 50 years first. The lower proportion of vaccinated patients reporting post-acute symptoms, and the lower proportion of vaccinated patients being hospitalized is also compatible with the existing literature^9, 13–15^. The most prevalent post-COVID symptoms in our cohort (supplementary Table s1) were similar to the symptoms of post-COVID condition frequently reported in the literature^6, 16^.

The study faced several limitations. Measured outcomes in the study were self-reported, therefore the possibility of reporting bias is a concern. In addition, our results are not generalisable to other COVID-19 vaccines as the population we reported on in this study were predominantly vaccinated with BNT162b2 vaccine and we did not determine which SARS-CoV-2 variant individuals were infected with. Furthermore, our study reports results from a cross-sectional study, therefore it was not possible to adequately compare the impact of COVID-19 vaccination on QoL over time. Consequently, caution should be taken while interpreting time trends results of COVID-19 vaccines presented in this study. The small numbers of individuals who received three doses and answered the survey more than 6 months post-infection was small, limiting the power of our dose-specific analysis. Finally, in the absence of a suggested minimally clinically important difference for this type of clinical presentation, it is difficult to extrapolate to what extent the changes in reported QoL scores translate clinically.

## Conclusions

Results from our study suggest that among individuals previously infected with SARS-CoV-2 virus, QoL in those unvaccinated at the time of infection was significantly lower than that in those vaccinated at the time of infection. COVID-19 vaccination, or at least vaccination with BNT162b2, can therefore modestly mitigate the decrease in QoL associated with symptoms of post-COVID illness, at least in the first 12 months. This mitigation could be largely explained by the reduction in severe acute illness associated with vaccination, but also by reducing the impact of post-acute COVID-19 symptoms on QoL. We could only detect positive associations between vaccination and QoL in those reporting up to 12 months following SARS-CoV-2 infection, but not beyond. Longitudinal studies are required to understand with more precision and certainty how symptoms post COVID-19 can affect QoL over time, and the role of vaccines and boosters in mitigating the long-term post-acute effects of SARS-CoV-2 infection. With Long COVID looking to become a durable public health issue affecting the quality of life of millions around the globe, studies estimating the MCID for Long COVID will help better understand the impact of interventions aimed at mitigating the effects of the disease.

## Methods

### Study design and participants

We invited individuals aged 18 years and older whose COVID-19 reverse transcription polymerase chain reaction (RT-PCR) test was done between 15th March 2020 and 15th June 2022 in one of three government hospitals in Northern Israel (Ziv Medical Centre, Padeh-Poriya Medical Centre, Galilee Medical Centre) to participate in the study. We included hospitalized patients and community patients whose PCR test was processed at a hospital laboratory. Participants recruitment and data collection has been described previously^14^. Briefly, using patient telephone records, eligible participants were invited to participate between July 2021 and June 2022, through a Short Message Service (SMS) with a link to an online survey available in four commonly spoken languages in Israel: Hebrew, Arabic, Russian, and English. In the current study, analysis was restricted to participants who reported having tested positive for SARS-CoV-2 by RT-PCR. We categorised participants according to the number of vaccine doses they received and then compared the groups according to their vaccination status in terms of reported QoL outcomes 3–18 months following their infection, both overall and among those reporting post-acute symptoms.

### Measurement tools

We used the International Severe Acute Respiratory and emerging Infection Consortium (ISARIC) COVID-19 follow-up tool^26^ and adapted it to the Israeli context. The questionnaire included the EQ-5D-5L tool, a widely used validated instrument for QoL measurement based on 5 dimensions: mobility, self-care, usual activities, pain/discomfort, and anxiety/depression. Each dimension is measured on a score scale from 1-(high QoL) to 5-(low QoL)^27^. A composite utility index (UI) was then generated, using country-specific weighting^28^. UI can range from 1 (complete health) to less than 0, acknowledging that extremely poor health statuses can lead to a QoL worse than death^29^.

### Data sources and variables

#### Baseline characteristics

Baseline characteristics recorded in the questionnaire included socio-demographics (marital status, age, sex, religion, ethnicity, and level of education), comorbidities (hypertension, diabetes, asthma, and COPD), and details about the acute COVID-19 episode including history of hospitalisation and intensive care admission.

#### Exposure groups

We categorised participants according to the number of COVID-19 vaccine doses received (0, 1, 2, or 3). In Israel, in the early phases of COVID-19 vaccination roll out, infected individuals were only eligible for a single dose. As a result, in our study, almost 90% of participants who received a single dose of vaccine were vaccinated after infection and were therefore not vaccinated at the time of infection. In the final analysis, we therefore grouped those who had received a single dose together with participants who reported not receiving any COVID-19 vaccine dose as a single group unvaccinated at the time of infection. Because Israel almost exclusively used the BNT162b2 vaccine in the study period, results apply to this vaccine only.

#### Assessment of symptoms of post-COVID disease

Participants were asked to select from a list of 39 symptoms which ones they were experiencing in the week prior to answering the survey. Participants who reported experiencing at least one of the ten most common symptoms were classified as experiencing post-acute COVID symptoms. To avoid misclassification between prolonged acute disease and post-acute symptoms, participants who reported symptoms in the first 60 days following their reported positive PCR test were excluded from the analysis.

#### Outcome: QoL

We measured participants’ QoL at the time of answering the survey using the EQ-5D-5L instrument^27^. The EQ-5D is a generic instrument for measuring quality of life. The instrument is based on a 5-level Likert scale descriptive system that measures health in 5 dimensions including: mobility, self-Care, usual activities, pain or discomfort, and anxiety/depression. Since no Israel-specific EQ-5D value set exists, following recommendations from the EuroQol Research Foundation, we computed the UI score using the USA EQ-5D value set.

### Statistical analysis

We described participant characteristics at baseline using means and standard deviations and proportions for continuous and categorical variables respectively. Two-sided t-tests were used to test the differences between group means and chi-square tests to compare proportions between groups. We computed the proportions of patients reporting specific scores for each of the 5 dimensions of the EQ-5D according to the number of vaccine doses received and presented the findings graphically. The mean QoL UIs with corresponding standard deviations (SDs) were computed for the included participants according to age, sex, ethnicity, vaccination status, time periods, education level, marital status, chronic illnesses status, and presence of post-COVID symptoms. We estimated the size of the effect of vaccination on Quality of life by calculating the standardized mean difference (Cohen’s d) between those vaccinated at the time of infection ( with 2 + doses) and those not (received 0 or 1 dose) among each stratum in our group according to age, gender, ethnicity, presence of post-acute symptoms and time since infection. In line with consensus thresholds we classified effect sizes as small (*d* = 0.2), medium (*d* = 0.5), and large (d ≥ 0.8).^30^.

We determined associations between vaccination status and post-COVID QoL using ordinary least square (OLS) linear regression, using a model adjusting for potential confounders. Variables considered in the model were those significant in the univariate analysis or deemed important in the literature and included time since SARS-CoV-2 infection (3–6 months, 7–12 months, and more than 12 months up to 18 months), number of COVID-19 vaccine doses received at the time of infection, presence of hypertension (the only underlying condition significantly different among those vaccinated and those not in the univariate analysis), age, sex, ethnicity and hospitalization. Because vaccination is associated with a reduction in severe disease^9^ and because severity of the acute episode is associated with post-COVID condition^25^, we ran the model both including and excluding hospitalization during the acute COVID-19 episode (as a proxy for disease severity), to determine whether any changes in QoL resulted from a decrease in acute disease severity or otherwise. We compared individuals vaccinated with 2 and 3 doses with individuals unvaccinated at the time of infection (either having received 0 doses or 1 dose after their infection) in terms of changes in reported QoL UI, together with 95% confidence intervals (95% CI). The regression analysis was then repeated stratified by the duration of time elapsed between vaccination and QoL reported: 3–6 months, 7–12 months, and 13–18 months. It is important to note that each time point includes different participants and we therefore do not directly test for trends over time.

### Ethical approval

The study was conducted in compliance with all relevant guidelines and regulations according to good clinical practice (GCP). All patients provided informed consent prior to participating in the study. The study was approved by the ethical committees of each of the three participating hospitals, namely Ziv Medical Centre, Padeh-Poriya Medical Centre, and Galilee Medical Centre ethical committees, reference numbers; 0007-21-ZIV, 009-21-POR, and 0018-21-NHR, respectively.

### Supplementary Information

Supplementary Tables.

## Data Availability

The dataset will be made available upon reasonable request to the authors. To request the dataset for secondary use please contact michael.edelstein@biu.ac.il.
