# Supplementary material for: Association between BNT162b2 vaccination and health-related quality of life up to 18 months post-SARS-CoV-2 infection in Israel
Source: Sci Rep. 2023 Sep 22;13:15801. doi: 10.1038/s41598-023-43058-1 (PMC10516916; doi:10.1038/s41598-023-43058-1)
Supplement: Supplementary file 1 — Supplementary Tables. [file 41598_2023_43058_MOESM1_ESM.docx]

**SUPPLEMENTARY TABLES**

s1. Frequency of most prevalent post-COVID symptoms in the cohort stratified by vaccination status of the respondent

| **Post-COVID symptoms** | **0-Doses** | **1-Dose** | **2-Doses** | **3-Doses** |
| --- | --- | --- | --- | --- |
| Fatigue | 82 (33·7) | 89 (38·5) | 79 (34·8) | 111 (44·4) |
| Headache | 75 (30·9) | 82 (35·5) | 71 (31·3) | 77 (30·8) |
| SOB | 31 (12·8) | 31 (13·4) | 34 (15·0) | 31 (12·4) |
| Chest pain | 29 (11·9) | 22 (9·5) | 25 (11·0) | 24 (9.6) |
| Pain on breathing | 20 (8·2) | 19 (8·2) | 21 (9·3) | 12 (4·8) |
| Palpitations | 33 (13·6) | 35 (15·2) | 31 (13·7) | 27 (10·8) |
| Loss of concentration | 35 (14·4) | 55 (23·8) | 43 (18·9) | 42 (16·8) |
| Altered taste | 16 (6·6) | 12 (5·2) | 17 (7·5) | 9 (3·6) |
| Altered smell | 25 (10·3) | 22 (9·5) | 24 (10·6) | 10 (4·0) |
| Persistent cough | 34 (14·0) | 36 (15·6) | 39 (17·2) | 45 (18·0) |
| Sleep disorders | 40 (16·5) | 50 (21·6) | 44 (19·4) | 47 (18·8) |
| Muscle pain | 43 (17·7) | 55 (23·8) | 46 (20·3) | 48 (19·2) |
| Abdominal pain | 22 (9·1) | 29 (12·6) | 26 (11·5) | 36 (14·4) |
| Constipation | 19 (7·8) | 15 (6·5) | 10 (4·4) | 24 (9·6) |
| Diarrhea | 17 (7·0) | 13 (5·6) | 16 (7·0) | 20 (8·0) |
| Difficulty urination | 5 (2·1) | 8 (3·5) | 5 (2·2) | 11 (4·4) |
| Hair loss | 47 (19·3) | 42 (18·2) | 37 (16·3) | 31 (12·4) |
| Joint pain | 29 (11·9) | 31 (13·4) | 22 (9·7) | 22 (8·8) |
| Dizziness | 37 (15·2) | 45 (19·5) | 43 (18·9) | 44 (17·6) |
| Weight loss | 8 (3·3) | 10 (4·3) | 12 (5·3) | 10 (4·0) |
| Nausea and vomiting | 14 (5·8) | 20 (8·7) | 23 (10·1) | 25 (10·0) |

s2. Proportions of participants reporting each level of EQ-5D-5L dimensions

| **EQ-5D-Level** | **Mobility** | | | | **Self-care** | | | | **Usual activities** | | | | **Pain and discomfort** | | | | **Anxiety and depression** | | | |
| --- | --- | --- | --- | --- | --- | --- | --- | --- | --- | --- | --- | --- | --- | --- | --- | --- | --- | --- | --- | --- |
|  | 0-doses | 1-dose | 2-doses | 3-doses | 0-doses | 1-dose | 2-doses | 3-doses | 0-doses | 1-dose | 2-doses | 3-doses | 0-doses | 1-dose | 2-doses | 3-doses | 0-doses | 1-dose | 2-doses | 3-doses |
| 1 | 165 (67·9) | 171 (74·0) | 178 (78·4) | 193 (77·2) | 206 (84·8) | 205 (88·7) | 211 (93·0) | 233 (93·2) | 162 (66·7) | 151 (65·4) | 130 (69·2) | 149 (74·4) | 149 (61·3) | 127 (55·0) | 130 (57.3) | 149 (59·6) | 152 (62·6) | 147 (63·6) | 155 (68·3) | 177 (70·8) |
| 2 | 37 (15·2) | 25 (10·8) | 20  (8·8) | 36  (14·4) | 19  (7·8) | 13  (5·6) | 5  (2·2) | 9  (3·6) | 28 (11·5) | 37 (16·0) | 5  (17·2) | 0 (16.8) | 14 (5·8) | 13 (5·6) | 5  (2·2) | 0  (0·0) | 14  (5·8) | 10  (4·3) | 7  (3·1) | 0  (0·0) |
| 3 | 17  (7·0) | 20  (8·7) | 14  (6·2) | 11  (4·4) | 8  (3·3) | 9  (3·9) | 6  (2·6) | 4  (1·6) | 32 (13·2) | 31 (13·4) | 73  (7·9) | 81  (3·6) | 53 (21·8) | 62 (26·8) | 73 (32·2) | 81 (32·4) | 53 (21·8) | 57 (24·7) | 57 (25·1) | 62 (24·8) |
| 4 | 19  (7·8) | 7  (3·0) | 10  (4·4) | 8  (3·2) | 8  (3·3) | 3  (1·3) | 3  (1·3) | 2  (0·8) | 18  (7·4) | 7  (3·0) | 15  (4·8) | 17  (4·4) | 16  (6·6) | 24 (10·4) | 15  (6·6) | 17  (6·8) | 16 (6·6) | 13  (5·6) | 6  (2·6) | 9  (3·6) |
| 5 | 5  (2·1) | 8  (3·5) | 5  (2·2) | 2  (0·8) | 2  (0·8) | 1  (0·4) | 2  (0·9) | 2  (0·8) | 3  (1·2) | 5  (2·2) | 4  (0·9) | 3  (0·8) | 11  (4·5) | 5  (2·2) | 4  (1·8) | 3  (1·2) | 8  (3·3) | 4  (1·7) | 2  (0·9) | 2  (0·8) |
| Total | 243 | 231 | 227 | 250 | 243 | 231 | 227 | 250 | 243 | 231 | 227 | 250 | 243 | 231 | 227 | 250 | 243 | 231 | 227 | 250 |

s3. Changes in utility indices for SARS-CoV-2 infected participants for all participants and for participants experiencing post-COVID symptoms, overall and by time elapsed since testing, not adjusted for hospitalization

| **Time elapsed since testing** | **Variables** | **All participants** | | | | **Participants experiencing post-COVID symptoms** | | | |
| --- | --- | --- | --- | --- | --- | --- | --- | --- | --- |
|  |  | **n** | **Adjusted** | | | **n** | **Adjusted analysis** | | |
|  | Vaccine doses |  | **Change in utility score** | **95% CI** | **P** |  | **Change in utility score** | **95% CI** | **P** |
| Overall | Unvaccinated at the time of infection | 471 | Baseline |  |  | 298 | Baseline |  |  |
|  | 2-Doses | 227 | 0·06 | 0·01 – 0·11 | **0**·**025** | 127 | 0·07 | <-0·01 – 0·14 | 0·059 |
|  | 3-Doses | 250 | 0·06 | -0·01 –0·12 | **0**·**050** | 147 | 0·08 | 0·02 – 0·15 | **0**·**016** |
|  | 2+3 Doses | 477 | 0·06 | 0·01 – 10·40 | **0**·**011** | 274 | 0·08 | 0·02 – 0·14 | **0**·**012** |
| 3-6 months | Unvaccinated at the time of infection | 118 | Baseline |  |  | 80 | Baseline |  |  |
|  | 2-Doses | 108 | 0·07 | -0·01– 0·15 | 0·088 | 53 | 0·04 | -0·05 – 0·15 | 0·545 |
|  | 3-Doses | 217 | 0·07 | <-0·01 – 0·13 | 0·051 | 128 | 0·09 | <0·01– 0·19 | **0**·**048** |
|  | 2+3 Doses | 323 | 0·07 | <0·01 – 0·13 | **0**·**036** | 181 | 0·08 | -0·01 – 0·16 | 0·086 |
| 7-12 months | Unvaccinated at the time of infection | 172 | Baseline |  |  | 109 | Baseline |  |  |
|  | 2-Doses | 48 | 0·09 | -0·01 – 0·20 | 0·087 | 35 | 0·15 | 0·02 – 0·29 | **0**·**021** |
|  | 3-Doses | 14 | 0·03 | -0·15 – 0·21 | 0·743 | 9 | <0·01 | -0·24 –0·24 | 0·996 |
|  | 2+3 Doses | 62 | 0·08 | -0·02 – 0·18 | 0·112 | 44 | 0·13 | <0·01 – 0·25 | **0**·**043** |
| >12 months | Unvaccinated at the time of infection | 184 | Baseline |  |  | 109 | Baseline |  |  |
|  | 2-Doses | 71 | 0·06 | -0·06 – 0·17 | 0·348 | 39 | 0·03 | -0·13 – 0·18 | 0·743 |
|  | 3-Doses | 19 | 0·03 | -0·16 – 0·22 | 0·755 | 10 | -0·20 | -0·28 – 0·24 | 0·873 |
|  | 2+3 D0ses | 90 | 0·05 | -0·06 – 0·16 | 0·362 | 49 | 0·02 | -0·13 – 0·16 | 0·830 |
| **Adjusted for: ethnicity, sex, age, and hypertension** | | | | | | | | | |
